# The Glass Ceiling for Women Managers: Antecedents and Consequences for Work-Family Interface and Well-Being at Work

**DOI:** 10.3389/fpsyg.2021.618250

**Published:** 2021-03-09

**Authors:** Audrey Babic, Isabelle Hansez

**Affiliations:** Human Resources Development Unit, Work Psychology Department, University of Liège, Liège, Belgium

**Keywords:** situational issues, interpersonal issues, organizational gender culture, differential treatment, glass ceiling, work-to-family conflict, well-being at work, organizational attitudes

## Abstract

Despite significant promotion of diversity in companies, as well as legislation for equal opportunities for women and men, it must be noted that women still remain largely in the minority in decision-making positions. This observation reflects the phenomenon of the glass ceiling that constitutes vertical discrimination within companies against women. Although the glass ceiling has generated research interest, some authors have pointed out that theoretical models have made little attempt to develop an understanding of this phenomenon and its implications. Therefore, our study aims to fill this gap and to better understand the phenomenon of the glass ceiling by considering both its antecedents and its possible consequences. More precisely, we extend the model developed by Elacqua et al. (2009), proposing a more comprehensive model including organizational gender culture as a third factor (in addition to situational and interpersonal issues) in the emergence of the glass ceiling through the perception of differential treatment. We also investigated the glass ceiling’s consequences for organizational attitudes and well-being at work by considering work-to-family conflict (WFC) as a possible mediator. We surveyed 320 women in managerial positions in a Belgian organization. Our study highlights the importance of all three factors in the emergence of the perception of differential treatment and, ultimately, the perception that a glass ceiling exists. Moreover, our results show that WFC fully mediates the effects of the glass ceiling on job strain and job engagement, and partially mediates the effects of the glass ceiling on job satisfaction and intention to quit.

## Introduction

In recent years, the number of women in the labor market has steadily increased (e.g., for Belgium^1^, 62.6% at the beginning of 2017 versus 67% at the beginning of 2019). This influx of women marks a change in mentality, giving rise to legal provisions and policies intended to guarantee them equal opportunities with men (Institut pour l’égalité des femmes et des hommes, 2013). Most of these procedures aim to facilitate the balance between family life and working life (flexible schedules, parental leave, and daycare, etc.; Institut pour l’égalité des femmes et des hommes, 2013). In Belgium, in 2011, the Council of Ministers passed a law imposing gender quotas in public organizations. Since January 2013, one third of companies’ boards of directors must be female. This approach promotes awareness of gender and management issues, as well as training for women managers to encourage them to apply for responsible positions.

However, despite these initiatives and the increasing number of qualified and trained women, it is clear that they are still largely underrepresented in the decision-making process in all sectors. Indeed, despite the current enthusiasm for diversity in companies and legislation for equal opportunities for women and men (e.g., the anti-discrimination law of May 10, 2007 and by extension, the gender law), the numbers have not changed much in the last decade. Women remain largely in the minority in decision-making positions. In 2018, women occupied 16% of positions on the executive committees of large Belgian companies:^2^ better than ten years ago, when the rate hovered between 9 and 10%, but still low compared to the overall employment rate of women and their share of the population of university graduates (60%).

These eloquent numbers illustrate the metaphor of the “glass ceiling.” This phenomenon of companies’ vertical discrimination against women (e.g., Cotter et al., 2001) has been widely studied by the academic community in various fields (management, human resources, finance, and psychology; e.g., Bell et al., 2002; Albrecht et al., 2003; Blau and Kahn, 2007). However, despite interest in this theme, theoretical models have made little attempt to develop an understanding of the “glass ceiling” and its implications (e.g., Cohen et al., 2020).

### Research Purpose and Objectives

In a previous study, Elacqua et al. (2009) developed a model in which beliefs about interpersonal and situational variables in the organization were related to the perception of differential treatment between men and women, which, in turn, was related to the perception of a glass ceiling. They demonstrated the importance of these two factors in the emergence of the glass ceiling through the perception of differential treatment. Indeed, moving into a company with few mentoring opportunities (i.e., interpersonal issue) and insufficiently objective evaluation criteria (i.e., situational issue) increased women managers’ perception of being treated differently from their male counterparts, leading therefore to the perception of glass ceiling.

However, these authors suggested furthering our understanding of this phenomenon by including other organizational variables that might be important to consider in the emergence of the glass ceiling. Thus, this study aims to enrich the model proposed by these authors by following their recommendation. Indeed, we considered a third factor as another antecedent to differential treatment, i.e., the organizational culture in relation to gender. We investigated if beliefs and stereotypes describing and prescribing social roles for male and female managers, in addition to interpersonal and situational issues, also increase women managers’ perception of being treated differently and therefore their perception of a glass ceiling.

Moreover, while Elacqua et al. (2009) focused their study on the causes of the glass ceiling, we want to extend the understanding of the phenomenon by also investigating its consequences for women managers’ organizational attitudes and well-being at work. Although some studies have demonstrated the deleterious effects of the glass ceiling on different concepts (e.g., intention to quit, Stewart et al., 2011; lower self-esteem, Tran, 2014; and a reduction in capability to build networks and support structures for one’s own career, Freeman, 1990), there remain some gaps in this domain, notably in terms of work-family interface. Indeed, to the best of our knowledge, no previous study has investigated the perception of work-to-family conflict (WFC) in association with the model proposed by Elacqua et al. Therefore, we investigated the consequences of women managers’ perception of a glass ceiling in terms of work-family interface and well-being (i.e., job strain, job engagement, job satisfaction, and intention to quit).

By investigating these issues, our study allows us to respond to specific recommendations and also fill some gaps in the literature on the glass ceiling. Therefore, through this research, we will try to better understand this phenomenon by considering both its antecedents and its possible consequences for well-being at work.

## Literature Review

### The Glass Ceiling

Glass ceiling refers to the fact that a qualified person whishing to advance within the hierarchy of his/her organization is stopped at a lower level due to a discrimination most often based on sexism or racism. The glass ceiling refers thus to vertical discrimination most frequently against women in companies. The difficulty inherent in this theme is the diversity of definitions and approaches describing the glass ceiling. There are also no objective and easily observable criteria that would make it possible to establish with certainty the real existence of a glass ceiling in a company. Nevertheless, based on several studies, the glass ceiling can be defined as subtle but persistent barriers/obstacles, underpinned by discriminatory, conscious and unconscious practices, and attitudes that hinder access to top/senior management positions for qualified women (e.g., Jackson and O’Callaghan, 2009; Bendl and Schmidt, 2010; Zeng, 2011). Glass ceiling refers thus to discriminatory barriers that prevent women from rising to positions of power or responsibility and advancing to higher positions within an organization simply because they are women (Li and Leung, 2001).

This phenomenon of the glass ceiling is based on several assumptions. Indeed, compared with other forms of discrimination and inequality, the glass ceiling is a particular and specific form of inequality due to several criteria (Cotter et al., 2001). First, the essence of the glass ceiling is the discrimination against women in management. The glass ceiling would therefore affect women despite their level of education, experience and skills. The glass ceiling is observed diachronically, i.e., it is advancements in women’s careers, promotions to managerial positions, that need to be taken into account, rather than the number of women in those positions at a specific time (Cotter et al., 2001). The glass ceiling also refers to the growing inequalities between men and women as they evolve in their professional careers within the company. Second, this bias is difficult to observe given that current equal opportunity policies prohibit open discrimination against these populations. Moreover, this includes norms/stereotypes revealed through practices, actions, facts, procedures, or attitudes that are frequently not directly observable. Finally, the existence of invisible barriers hinders hierarchical ascension (Cotter et al., 2001). Indeed, this definition focuses on top/senior management, with the assumption that the glass ceiling occurs more frequently at this level than at middle and lower grades. These obstacles to advancement are more present as one approaches the top of the hierarchy (Cotter et al., 2001). Indeed, most researchers admit that the singularity of this phenomenon lies in its predominance at higher levels of management (Baxter and Wright, 2000; Albrecht et al., 2003; Elliott and Smith, 2004; Prokos and Padavic, 2005; Zeng, 2011; Dambrin and Lambert, 2012; Lupu, 2012).

### The Antecedent of the Glass Ceiling: The Model of Elacqua et al. (2009)

A model for understanding the phenomenon of the glass ceiling is that developed by Elacqua et al. Through their study, these authors investigated why women managers rarely reach the highest levels of their organization. Among 685 managers at a large Midwestern insurance company, they proposed a model in which beliefs about organizational variables of an interpersonal and situational nature were positively related to the perceptions of differential treatment between men and women, which, in turn, was positively related to the perception of a glass ceiling. Therefore, these authors suggest that perceptions of differential treatment mediate the relationships between both these organizational factors (i.e., interpersonal and situational issues) and perceptions of a glass ceiling.

#### Interpersonal Factors

According to these authors, some interpersonal relationships can influence how women and their male counterparts are treated differently within the company. In their study, Elacqua et al. examined in particular: (a) mentoring, (b) the existence of an informal network of senior managers, and (c) the friendly relationships with company decision-makers, as these concepts are all related to career advancement. Indeed, regarding the first of these three aspects, research has highlighted that a lack of high-level organizational mentors is deleterious to women’s career progression, especially since mentoring is an important source of information (e.g., Ibarra et al., 2010). In addition, mentor-supported individuals perceived more opportunities for promotion (Allen et al., 2004), and the likelihood that they will actually be promoted is greater (Dreher and Ash, 1990; Allen et al., 2004). These people would also be more satisfied with their careers and work (Kammeyer-Mueller and Judge, 2008). Thus, mentoring would be a significant enabler in women’s career advancement and even business development (Elkin, 2006). As mentioned by Elacqua et al. (2009, p. 286), “employees whose supervisors act as their mentors are more likely to feel that they are not excluded from important information and opportunities, and, therefore, assume that is true for others as well.” As a result, they perceive less differential treatment among employees in their company (Raabe and Beehr, 2003).

The second aspect of interpersonal factors investigated by the authors is the existence of an informal social network of senior men within the firm. Networks refer to the development and use of career-relevant contacts in which members exchange valuable strategic information (concerning new positions, ongoing projects, and managerial decisions, etc.), contacts and recommendations (Burke, 1984). Several studies have shown that women are being assigned positions with lower visibility, limiting their opportunities to connect with high-ranking individuals and develop social networks (e.g., Ragins et al., 1998). In companies where there is an informal social network of senior men, women managers may not be treated in the same way as men because of lack of visibility (Elacqua et al., 2009). Limited access to such a network would reduce the chances of promotion and therefore, lead to a perception of a glass ceiling (Brass, 1985).

The third aspect of interpersonal factors refers to the friendly relationships with the company’s decision-makers. Individuals often like to form friendships with people of the same sex who have had similar experiences. Women managers might then face an additional difficulty: the “queen bee syndrome” (Keeton, 1996). This syndrome describes the fact that some women managers who have managed to get to the top feel that they have had to work hard to get where they are. They think that other women should work as hard to succeed. Indeed, according to Cech and Blair-Loy (2010), women breaking the glass ceiling tend to attribute their success to merit rather than to overcoming the structural barriers that senior women are able to do something about.

In their study of a large U.S. sample of female accountants, Cohen et al. (2020) highlighted the importance of these interpersonal factors in the occurrence of the glass ceiling. Indeed, they found that a lack of mentoring opportunities, networking opportunities, social support from male organizational leaders and high-profile job assignments have a strong positive influence on female accounting professionals’ glass ceiling perceptions.

#### Situational Factors

Regarding these second kind of factors, Elacqua et al. considered two aspects that would influence the perception of a glass ceiling through the perception of differential treatment. The first aspect is the existence of objective criteria for procedures established within the company (e.g., hiring, and promotion). Lyness and Heilman (2006) have shown that the promotion criteria are more severe and more related to job performance for female line managers compared to their male colleagues. Women are particularly sensitive to and supportive of being promoted on the basis of their performance (Beehr et al., 2004). Employees who believe that their company uses objective criteria related to skills and performance for the promotion process rate the process as fairer and do not perceive differential treatment among employees (Beehr et al., 2004). In their study, Cohen et al. (2020, p. 22) also found that “female accounting professionals who believe that female employees are not treated the same as male accounting professionals with regard to performance evaluations will be more likely to report a glass ceiling within their organizations.”

The second aspect refers to “the number of women managers who have been in managerial positions long enough to be considered serious candidates for advancement to higher levels” (Elacqua et al., 2009, p. 287). According to Elacqua et al., one would think that a woman manager is a serious candidate for a promotion if she already occupies a managerial position and participates in the development activities of the company. However, as mentioned by Elacqua et al. (2009, p. 287), “women experience these two situations less frequently than men.” Therefore, there will not be as many women in position for promotion to higher-level management positions (Elacqua et al., 2009). “If managers believe that this is happening in their organization, they are likely to perceive differential treatment of sexes and consequently the existence of a glass ceiling” (Elacqua et al., 2009, p. 287).

#### Perception of Differential Treatment of Women

Men and women are often treated differently in the world of work (Blau and Kahn, 2007; Kochan, 2007). These disparities arise when “personnel decisions are based on gender, an ascribed characteristic, rather than on an individual’s qualifications or job performance (Gutek et al., 1996; Ngo et al., 2002)” (cited by Foley et al., 2005, p. 423). According to Elacqua et al., perceiving differences in a company’s treatment of women would lead workers to believe that there is a glass ceiling in the company. While gender differences may be relatively small in terms of, for example, promotion at each level of the hierarchy, they add up to form a gap between the number of men and women occupying the highest positions in the company (Agars, 2004).

#### Organizational Culture in Relation to Gender

In their study, Elacqua et al. suggested further investigation of other factors that may influence the perception of differential treatment of men and women and of a glass ceiling. Indeed, according to these authors, “glass ceilings and perceptions of it are especially likely to be found in cultures that encourage differential views of and treatment of men and women” (Elacqua et al., 2009, p. 293). Another factor influencing the emergence of the glass ceiling through the perception of differential treatment could be the culture implemented within organizations, in other words the organizational culture, referring to organizationally shared values and beliefs that reflect workers’ judgments of how things should be and really are (Lord and Maher, 1991). The literature has shown that two main aspects of organizational culture in relation to gender are important barriers to women’s progress (e.g., Eagly and Johnson, 1990; Valian, 1998), i.e., the “male-oriented” organizational culture and beliefs about the incompatibility of the roles of mother, wife and manager. Both these aspects refer to the set of beliefs and stereotypes describing and prescribing social roles for male and female managers that are conveyed by the organization and some of its members.

Senior managers, overwhelmingly men, define a “gendered” culture that excludes and marginalizes women. This culture consists of a series of norms and organizational practices that promote and define values, stereotypes, behaviors, and a vision of management and leadership that are “masculine” (van Vianen and Fisher, 2002; Broadbridge and Hearn, 2008; Koenig et al., 2011). In line with the social role theory (Eagly, 1987), the image of the manager is often associated with that of a man with so-called “masculine” qualities, such as authority, independence, competitiveness, and aggressiveness (e.g., Weyer, 2007). Therefore, women, who have been associated for centuries with diametrically opposing qualities (e.g., collaboration, listening, sensitivity, and sympathy), would be less committed to their careers and unable to manage (e.g., Weyer, 2007). These gender stereotypes about women have an adverse impact on their assessments and judgments (Lyness and Thompson, 1997; van Vianen and Fisher, 2002). As a result, early in their careers, women managers are assigned different responsibilities from those of men. Women managers are then faced with a twofold constraint: (a) if they do not conform to male norms, they risk being judged and evaluated negatively; and (b) if they adopt a “masculine” attitude, they get hurt by their colleagues (Oakley, 2000; Eagly and Karau, 2002; Mavin, 2008; Kumra and Vinnicombe, 2010).

Other beliefs about women managers can lead their superiors to fail to consider them as serious candidates for top management positions. Among these beliefs are the incompatibility of the roles of mother, wife and manager; being a manager requiring too much investment, flexibility and travel. In line with this idea, Hoobler et al. (2008) have shown that managers think that work-life conflict is greater for women than for their male counterparts. However, this belief has implications since it will reduce the organization’s perception of women’s adequacy in the workplace, decreasing the probability of female promotion (Rudman and Phelan, 2008).

Therefore, based on the above, we postulate that perceptions of interpersonal issues, situational issues and the organizational gender culture will influence perceptions of differential treatment, which in turn, will affect the perception of a glass ceiling:

*Hypothesis 1a*: Perceptions of differential treatment will mediate the relationships between interpersonal issues and the perception of a glass ceiling.

*Hypothesis 1b*: Perceptions of differential treatment will mediate the relationships between situational issues and the perception of a glass ceiling.

*Hypothesis 1c*: Perceptions of differential treatment will mediate the relationships between the organizational gender culture and the perception of a glass ceiling.

### Consequences of the Glass Ceiling

Although there are many articles on the glass ceiling, very few, to our knowledge, and investigate its effects on workers. That is the reason why one of the objectives of our study is to extend the understanding of this phenomenon by also investigating its consequences for organizational attitudes and women managers’ well-being. Better assessing the consequences of the glass ceiling seems essential to sensitize companies to this issue. Through our study, we want to investigate the links between the perception of the glass ceiling and five concepts, i.e., WFC, job strain, intention to quit, job engagement, and job satisfaction. Specifically, we intend to investigate the mediating role of WFC in the relationship between the glass ceiling and the other four outcomes. We have included job strain and intention to quit in this study because they are the strongest outcomes related to WFC among work-related outcomes (Allen et al., 2000; Amstad et al., 2011), and job satisfaction because it is the work-related outcome that has attracted the most research attention (Allen et al., 2000; Amstad et al., 2011). Considering that little is known about the effects of WFC on positive indicators of well-being (Peeters et al., 2013), we have considered job engagement in relation with WFC because this concept is a well-known indicator of well-being (Bakker and Demerouti, 2008, 2017).

#### Relationship Between the Glass Ceiling and WFC

Individuals must daily take on several social roles (i.e., parent, spouse, and employee, etc.). Contradictory demands can arise from these multiple social roles in which individuals have to perform. The force to comply with these contradictory demands can produce incompatibilities between professional and family roles (Duxbury and Higgins, 1991). Indeed, given that individuals have limited resources notably in terms of time and energy, they can not satisfy all of these demands (scarcity hypothesis, Sieber, 1974). Therefore, when individuals have to compose with too many demands arising from work, they may experience WFC defined as “a form of inter-role conflict in which the general demands of time devoted to, and strain created by the job interfere with performing family-related responsibilities” (Netemeyer et al., 1996, p. 401).

To the best of our knowledge, no previous study has investigated the link between the glass ceiling and WFC. However, we can argue that the glass ceiling increases the perception of WFC. One theory for postulating this link is the conservation of resources theory (COR theory, Hobfoll, 1989, 2001, 2011). According to this theory, individuals and groups are threatened by the potential or actual loss of that which they value highly, namely resources. Therefore, people are motivated to obtain, acquire, retain, preserve, protect, foster, and expand valued resources for anticipated future needs (Hobfoll, 1989). Indeed, as mentioned by Hobfoll (2011, p. 117), people “employ key resources in order to conduct the regulation of the self, their operation of social relations, and how they organize, behave, and fit in to the greater context of organizations and culture itself.” Within this theory, resources are crucial and refer to “those objects, personal characteristics, conditions, or energies that are valued by the individual or that serve as a means for attainment of these objects, personal characteristics, conditions, or energies” (Hobfoll, 1989, p. 516).

We can reasonably think that, in perceiving a glass ceiling, women managers have access to fewer resources at work (e.g., little access to information, advice, social support, supervisory coaching, opportunity to develop knowledge, performance feedback, and promotion). The glass ceiling also undermines personal characteristics (e.g., self-esteem, self-efficacy, and optimism) and energies (e.g., knowledge, and money). Indeed, women facing such a form of discrimination are disadvantaged with regard to choice of job, salary, and prestige (Baxter and Wright, 2000). Perceptions of a glass ceiling also inhibit women from seeking and obtaining promotions (Powell and Butterfield, 1994), and reduce their self-esteem (Tran, 2014) and their capabilities to build networks and support structures for their own careers (Freeman, 1990). As mentioned by Downes et al. (2014, p. 133), “women also internalize negative evaluations and stereotypes by those in the majority to the point where they limit themselves and turn down opportunities for advancement due to the fear that they will not succeed (Ilgen and Youtz, 1986).”

It is also possible that women managers, in order to fight or override barriers/obstacles related to the glass ceiling, invest resources to try to progress in the hierarchy but that this investment is not effective. Indeed, using resources to cope with a situation of resource loss is also stressful because this may deplete an individual’s stock of resources (Hobfoll, 1989). As mentioned by Hobfoll, in situations where “resources expended in coping outstrip the resultant benefits, the outcome of coping is likely to be negative” (Hobfoll, 1989, p. 518).

As a result, when there is a loss of resources and when no action is taken or when resource investment is unsuccessful, a spiral of loss of resources appears in which more and more resources are lost. In other words, initial loss of resources produces further loss (Hobfoll, 2001). This spiral contributes to poor psychological and/or physical health. These negative consequences (e.g., negative emotions, impaired psychological well-being, and ultimately impaired mental and physical health) spill over and negatively affect individuals’ functioning at home (Edwards and Rothbard, 2000), increasing the perception of WFC.

#### Relationship Between WFC and Job Strain

The imbalance in professional and family roles is an important stressor, influencing work, and private domains and affecting health and well-being in general (Frone, 2003).

According to the COR theory, stress is defined as “a reaction to the environment in which there is (a) the threat of a net loss of resources, (b) the net loss of resources, or (c) a lack of resource gain following the investment of resources. Both perceived and actual loss or lack of gain are envisaged as sufficient for producing stress” (Hobfoll, 1989, p. 516). Losing resources, or the threat of such a loss, may cause stress and strain. Applied to work–family conflict, Grandey and Cropanzano (1999, p. 352) mentioned that “inter-role conflict leads to stress because resources are lost in the process of juggling both work and family role.” Supporting this theoretical view, considerable empirical research has shown that the WFC is positively related to job strain (e.g., meta-analyses of Allen et al., 2000 and Amstad et al., 2011). For example, in their study on firefighters in Taiwan, Wong et al. (2014) found a positive relationship between WFC and job strain. In studies of employees of a Belgian hospital (Babic et al., 2015) and of employees of a Belgian company (Babic et al., 2019), WFC was also found to be positively related to job strain.

#### Relationship Between WFC and Intention to Quit

According to the COR theory, “because resource loss is stressful and because people must invest resources to offset further resource loss, once initial losses occur, people become increasingly vulnerable to ongoing loss” (Hobfoll, 2001, p. 355). Moreover, as mentioned by Vinokur-Kaplan (2009, p. 231), “because people have fewer resources as they lose resources, they are decreasingly capable of withstanding further threats to resource loss.” This situation ultimately results in a loss spiral in which more and more resources are lost. Facing such situations of resources loss, in order to protect the remaining resources, people perceiving WFC want to flee this situation (Hobfoll, 1989) and leave their organization (e.g., Greenhaus et al., 1997). Empirical research has supported this theoretical view. The meta-analyses conducted by Allen et al. (2000) and Amstad et al. (2011) showed that intention to leave the organization significantly related to WFC. Through a study on US workers, Anderson et al. (2002) found that perceiving WFC increased workers’ turnover intention. Hammer et al. (2009) also found a positive relationship between these constructs. On 197 Maori employees working in 13 New Zealand organizations in diverse geographical locations, Haar et al. (2012) also found that WFC was significantly related to turnover intentions. Through studies of 509 employees of a Belgian hospital, Babic et al. (2015) found a positive relationship between WFC and intention to quit.

#### Relationship Between WFC and Job Engagement

As previously mentioned, workers losing resources due to WFC (Hobfoll, 1989) want to protect their remaining resources (Hobfoll, 2002). Another way for workers to protect their resources is to reduce their level of engagement in their work. Moreover, in line with the source attribution perspective of WFC (Shockley and Singla, 2011), workers perceiving that their work interferes negatively with their family sphere blame their professional domain given that it is the source of the perceived conflict. When facing such situations of resource loss or dissatisfaction, workers react or cope by adjusting their attitudes (e.g., reducing their engagement in their work). Supporting these theoretical views, empirical research has shown that the WFC employees have to deal with can affect their job engagement (e.g., Wilczek-Ruzyczka et al., 2012; Opie and Henn, 2013; Fiksenbaum, 2014). Through their study on 267 South Africans working, Opie and Henn (2013) found that employees experiencing WFC engaged less in their work. In a sample composed of 978 workers from a Belgian Federal Public Service, Babic et al. (2017) found that WFC had a negative impact on work engagement. Through their study of 98 nurses from southern Poland, Wilczek-Ruzyczka et al. (2012) found that, compared to nurses perceiving lower WFC, those perceiving higher WFC engaged less in their work (lower level of vigor and dedication). In a sample of employees of a Belgian Federal Public Service, Babic et al. (2020) also found that WFC was negatively associated with vigor.

#### Relationship Between WFC and Job Satisfaction

According to the source attribution approach (Shockley and Singla, 2011), in perceiving WFC, individuals are dissatisfied with the work domain because they psychologically blame the domain that is the source of conflict (i.e., work). Therefore, when work causes difficulties in fulfilling family responsibilities, individuals obtain less satisfaction from work (e.g., Höge, 2009). Empirical research has supported this theoretical view. Through their study on US workers, Anderson et al. (2002) found that WFC was negatively related to job satisfaction. Through their study on 360 employees from 12 stores of a grocery store chain in the Midwestern United States, Hammer et al. (2009) found a similar result. Carlson et al. (2010) also found that perceiving WFC leads workers to have lower level of job satisfaction. In a study of 509 employees of a Belgian hospital, Babic et al. (2015) found a negative relationship between WFC and job satisfaction. This negative relationship was also highlighted by several meta-analyses (e.g., Kossek and Ozeki, 1998; Allen et al., 2000; Amstad et al., 2011; Shockley and Singla, 2011).

Therefore, based on the above, we postulate that the perception of a glass ceiling will increase the perception of WFC, which, in turn, will increase job strain and intention to quit, and decrease job engagement and job satisfaction:

*Hypothesis 2a*: WFC will mediate the relationships between the glass ceiling and job strain.

*Hypothesis 2b*: WFC will mediate the relationships between the glass ceiling and intention to quit.

*Hypothesis 2c*: WFC will mediate the relationships between the glass ceiling and job engagement.

*Hypothesis 2d*: WFC will mediate the relationships between the glass ceiling and job satisfaction.

**FIGURE 1:**
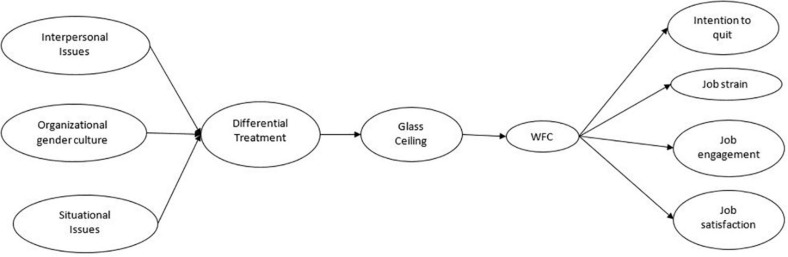
Hypothesized theoretical model. WFC, work-to-family conflict.

Figure 1 depicts the hypothesized theoretical model.

## Materials and Methods

### Sample

The organization in which our study was conducted is a global healthcare company. In terms of culture and value, this company is careful to treat its employees with respect and in an equal manner. This company aims to create an inclusive, engaging/stimulating work environment that encourages employees to get involved to help achieve company goals. To do so, the company offers all of its employees a range of learning opportunities, training and professional development programs. On-the-job learning is encouraged through project-based work, while providing support through more traditional training programs, mentoring and coaching. In terms of learning opportunities, the organization, for example, sets up feedback from the line manager and peers. Regular meetings with managers are also set up in order to plan development objectives. These meetings focus on the development aspirations, skills, experience, and needs of workers. This company is attentive to the well-being of its workers and to their possibility to balance their professional and private life. For example, the organization offers benefits such as access to health care and wellness programs or joining a retirement plan.

The company invests heavily in the development of its employees at all stages of their careers, from young graduates to senior managers. In terms of inclusion and diversity, the company designs workshops to sensitize and empower employees to create a workplace free from discrimination. Different campaigns have been put in place to recognize and manage unconscious prejudices with regard to gender, age, sexual orientation, gender identity, ethnicity, or even religion.

In order to test our hypotheses, a self-reported questionnaire was administered to women occupying managerial positions within this global healthcare company. One thousand two hundred thirty women managers were invited to participate in this research. We received 320 questionnaires in return, corresponding to a response rate of about 26%. Twenty-six percent of respondents were between 36 and 40 years old. Forty percent had been employed by their company for between 2 and 5 years. Eighty-one percent were either married or living with a partner. Seventy percent had one child or more at home. Fifty-six percent led a team. Sixty-six percent occupied a first line management position, thirty-one percent occupied a middle management position, and only three percent were part of senior management.

Using the full partial covariate effects (Little, 2013), three socio-demographic variables (age, grade, and presence of children at home) were significantly related with the constructs of our model. Consequently, we included these three socio-demographic variables as covariates to control for their effects in our analyses.

### Procedure

This study was carried out in accordance with the recommendations of the APA Ethical Principles of Psychologists and Code of Conduct with written informed consent from all subjects. All subjects participated in a free and informed manner and gave written informed consent in accordance with the Declaration of Helsinki. Data were collected through an online survey. Participants received an e-mail explaining the purpose of the study and emphasizing the confidentiality of the responses (anonymous participation). The informed consent of each participant was obtained. People were given 1 month to complete the survey that was anonymous and confidential. As participants speak French and English, questionnaires were written in these two languages. Lacking all the scales in these two languages, we translated them following a translation back-translation procedure (Brislin, 1980). For all scales, there was no major discrepancy between the original and translated versions, so the translation process was considered appropriate.

### Measures

*The glass ceiling and its antecedents* were assessed using the questionnaires developed by Elacqua et al. (2009). To develop their questionnaire, Elacqua and her colleagues had established a task force composed of employees of the company in which they carried out their study and an industrial/organizational psychologist with expertise in survey research. This task force developed a series of items relating to the different variables the authors wanted to measure. These items reflected the company’s concerns about gender equality at work. However, these items were developed in reference to an American culture. Therefore, in order to have items more in line with European culture, we added to the items of Elacqua et al. some questions created in collaboration with the company in which this study was carried out (see Supplementary Appendix 1 for the overall questionnaire). All items (original and new) were higher than 0.50, which is the recommended cut-off score for factor loadings (Kline, 2011). All were measured using a 4-point Likert-type rating scale ranging from “strongly disagree” to “strongly agree.” For all of the scales, a high score indicates discrimination against women managers. *Interpersonal issues* were assessed with the four original items developed by Elacqua et al. (e.g., “Having a personal friendship with the decision-makers determines whether an employee will be considered for a promotion at my company.”) and with four new items (e.g., “Few women managers have access to strategic information coming from the senior management”). Cronbach’s alpha was 0.75. *Situational issues* were assessed with the three original items developed by Elacqua et al. (e.g., “There are few women at the top rung of management at my company because they haven’t been in the management ranks long enough”) and with three new items (e.g., “In my company, promotion procedure is based on objective criteria”). Cronbach’s alpha was 0.72. *Organizational gender culture* was evaluated with four new items (e.g., “My colleagues and superiors estimate that women managers are less mobile and flexible than their male counterparts”). Cronbach’s alpha was 0.70. *Differential treatment* was evaluated with the five original items developed by Elacqua et al. (e.g., “At my company, there are differences in salaries and titles for men and women in the same positions that are not explained by differences in performance, education, experience”) and with two new items (e.g., “In my company, performance criteria are different for men and women managers”). Cronbach’s alpha was 0.71. *Perceptions of a glass ceiling* were evaluated with the three original items developed by Elacqua et al. (e.g., “Female managers/supervisors at my company generally progress to a certain level, then go no further”) and with three new items (e.g., “In my company, with equal experience and expertise, men have access to higher positions in the hierarchy than women”). Cronbach’s alpha was 0.84. We performed an exploratory factor analysis and found that both new items and those developed by Elacqua et al. loaded adequately on their respective factors. The full measurement model concerning these 31 items are presented in the Supplementary Appendix 2.

*Work-to-family conflict* was assessed using the appropriate SWING subscale (Geurts et al., 2005). This nine-item subscale evaluates the negative impact of the professional situation on family life (e.g., “I’m irritable at home because my work is demanding”). People responded on a 4-point Likert-type scale (0: never to 3: always). Cronbach’s alpha was 0.87.

*Job strain* was measured with the Negative Occupational State Inventory subscale developed by Barbier et al. (2012). This scale is comprised of nine items (e.g., “My work stresses me”). People responded on a 4-point Likert-type scale (1: never to 4: always). Cronbach’s alpha was 0.78.

*Job engagement* was measured with the Positive Occupational State Inventory subscale developed by Barbier et al. (2012). This scale included eight items (e.g., “When I’m working I forget my tiredness”). People responded on a 4-point Likert-type scale (1: never to 4: always). Cronbach’s alpha was 0.81.

*Job satisfaction* was measured with the scale used by Eisenberger et al. (1997). This scale comprised four items (e.g., “All in all, I am very satisfied with my current job”). People responded on a 5-point Likert-type scale (1: strongly disagree to 5: strongly agree). Cronbach’s alpha was 0.87.

*Intention to quit* was estimated using Hom and Griffeth’s (1991) scale which comprises three items (e.g., “I often think about quitting my organization”). People responded on a 5-point Likert-type scale (1: strongly disagree to 5: strongly agree). Cronbach’s alpha was 0.92.

### Data Analyses

Structural equation modeling analyses were performed using Lisrel 8.80 (Jöreskog and Sörbom, 2006). Data were analyzed following a two-stage process suggested by Anderson and Gerbing (1988). First, we assessed the measurement model through a series of confirmatory factor analyses to evaluate the independence of constructs examined in our study. Second, we proceeded with the assessment of the hypothesized structural relationships among latent variables. For this second stage, in order to limit the number of parameters to be estimated, we reduced the number of items per factor by combining them to create a limited number of indicators per construct (Landis et al., 2000). Using the balancing technique, we generated aggregate indicators by averaging items with high and low loadings. We used this technique in order to have parcels equally balanced in terms of their discrimination (Little et al., 2002). We thus reduced the number of items to three for each of our constructs. We decided to use parceling strategy for different reasons. Considering psychometric characteristics, parcels have higher reliability, greater communality, a higher ratio of common to unique factor variance, a lower likelihood of distributional violations, and more-equal intervals compared to item-level analyses (Little et al., 2013). Moreover, considering model estimation and fit characteristics, parcels have fewer parameter estimates, lower indicator-to-sample size ratio, a lower likelihood of correlated residuals and dual factors loading and reduces sources of sampling errors (Little et al., 2013).

## Results

### Discriminant Validity

We tested the distinctiveness between the variables included in our study by comparing several nested models (Bentler and Bonnett, 1980). First, we examined the fit of our hypothesized ten-factor model (i.e., interpersonal issues, situational issues, organizational gender culture, differential treatment, the glass ceiling, WFC, job strain, job engagement, job satisfaction, and intention to quit). The results indicate that this hypothesized measurement model fit the data reasonably well [χ^2^(360) = 628.79, *p* < 0.001, RMSEA = 0.05, NNFI = 0.97, and CFI = 0.98]. Moreover, loadings of all items, including new items created to assess the glass ceiling and its antecedents, were higher than 0.50, which is the recommended cut-off score for factor loadings (Kline, 2011).

Starting from this ten-factor model, we tested a series of more constrained measurement models. In particular, we tested ten nine-factor models, one eight-factor model, one seven-factor model, and one six-factor model obtained by combining the glass ceiling and its antecedents. Chi-square difference tests were used to compare the fit of these nested models with that of the ten-factor model (Bentler and Bonnett, 1980). Results indicated that the ten-factor model was significantly superior to all alternative models. Consequently, we used this ten-factor model to test our hypotheses. Table 1 displays fit indices for some of these alternative models.

**TABLE 1:** Fit indices for measurement models.

N°	Model	Df	χ ^2^	RMSEA	NNFI	CFI	COMPARISON	Δχ ^2^ (Δ df)
1	10-factor model	360	628.79	0.05	0.97	0.98	—	—
2	9 factors (situ with cult)	369	841.28	0.06	0.95	0.96	1 vs 2	212.49 (9)***
3	9 factors (situ with inter)	369	789.96	0.06	0.96	0.96	1 vs 3	161.17 (9)***
4	9 factors (cult with inter)	369	748.69	0.06	0.96	0.97	1 vs 4	119.9 (9)***
5	9 factors (situ with treat)	369	756.93	0.06	0.96	0.97	1 vs 5	128.14 (9)***
6	9 factors (cult with treat)	369	771.41	0.06	0.96	0.97	1 vs 6	142.62 (9)***
7	9 factors (inter with treat)	369	672.56	0.05	0.97	0.97	1 vs 7	43.77 (9)***
8	9 factors (situ with gc)	369	757.46	0.06	0.96	0.97	1 vs 8	128.67 (9)***
9	9 factors (cult with gc)	369	758.68	0.06	0.96	0.97	1 vs 9	129.89 (9)***
10	9 factors (inter with gc)	369	696.77	0.05	0.97	0.97	1 vs 10	67.98 (9)***
11	9 factors (treat with gc)	369	678.02	0.05	0.97	0.97	1 vs 11	49.23 (9)***
12	8 factors (inter with situ with cult)	377	919.09	0.07	0.95	0.95	1 vs 12	290.30 (17)***
13	7 factors (inter with situ with cult with treat)	384	951.97	0.07	0.95	0.95	1 vs 13	323.18 (24)***
14	6 factors (inter with situ with cult with treat with gc)	390	993.23	0.07	0.94	0.95	1 vs 14	364.44 (30)***

N = 320. situ, situational issues; cult, organizational gender culture; inter, interpersonal issues; treat, differential treatment; gc, glass ceiling; df, degrees of freedom; χ^2^, Minimum Fit Function Chi-Square; RMSEA, root-mean-square error of approximation; NNFI, Non-Normed Fit Index; CFI, Comparative Fit Index; and Δχ^2^, chi-square difference tests between the ten-factor model and alternative models. ***p < 0.001.

#### Relationships Among Variables

Means, standard deviations, Cronbach’s alphas and correlations among variables are presented in Table 2. Internal consistency reliabilities ranged from 0.70 to 0.92.

**TABLE 2:** Descriptive statistics and inter-correlations among variables.

Variables		*M*	SD	1	2	3	4	5	6	7	8	9	10	11	12	13
1	Age	–	–	–												
2	Grade	–	–	0.38***	–											
3	Children at home	–	–	0.24***	0.06	–										
4	Interpersonal issues	2.87	0.70	0.12*	−0.04	−0.02	(0.75)									
5	Situational issues	3.08	0.64	−0.09	0.02	0.05	−0.19***	(0.72)								
6	Organizational of gender culture	2.82	0.84	0.08	−0.04	0.07	0.39***	−0.10	(0.70)							
7	Differential treatment	2.63	0.62	0.02	0.01	−0.02	0.45***	−0.17**	0.42***	(0.71)						
8	Glass ceiling	2.82	0.81	0.07	0.00	0.00	0.56***	−0.26***	0.49***	0.56***	(0.84)					
9	WFC	2.17	0.52	0.11	0.05	0.11	0.19***	−0.09	0.21***	0.11*	0.19***	(0.87)				
10	Job strain	1.85	0.52	0.01	−0.05	0.00	0.20***	−0.11*	0.20***	0.15**	0.17**	0.38***	(0.78)			
11	Job engagement	3.10	0.59	−0.07	0.07	0.02	−0.21***	0.11*	−0.11*	−0.05	−0.20***	−0.15**	−0.20***	(0.81)		
12	Job satisfaction	3.80	0.81	−0.10	0.00	−0.05	−0.36***	0.28***	−0.21***	−0.21***	−0.35***	−0.35***	−0.33***	0.45***	(0.87)	
13	Intention to quit	2.12	0.97	−0.04	−0.03	−0.15**	0.34***	−0.27***	0.24***	0.24***	0.37***	0.22***	0.25***	−0.32***	−0.53***	(0.92)

N = 320. Correlations among variables are provided below the diagonal and Cronbach’s alphas are provided on the diagonal. Cult, organizational gender culture; WFC, work-to-family conflict. *p < 0.05; **p < 0.01; and ***p < 0.001.

Based on the results of the confirmatory factor analyses, we examined the structural relationships among latent variables through a series of alternative models (Models 2 to 8). Table 3 presents the fit indices for these alternative models. Model 1 (i.e., the hypothesized model) fit the data reasonably well [χ^2^(468) = 912.36, *p* < 0.001, RMSEA = 0.06, NNFI = 0.96, and CFI = 0.96].

**TABLE 3:** Fit indices for structural models.

Model	df	χ ^2^	RMSEA	NNFI	CFI	COMPARISON	Δχ ^2^ (Δ df)
Model 1: Hypothesized theoretical model	468	912.36	0.06	0.96	0.96	—	—
Model 2: Model 1 + Paths between inter and gc	467	911.74	0.06	0.96	0.96	Model 1 vs Model 2	0.62(1)
Model 3: Model 1 + Paths between situ and gc	467	910.38	0.06	0.96	0.96	Model 1 vs Model 3	1.98(1)
Model 4: Model 1 + Paths between cult and gc	467	909.36	0.06	0.96	0.96	Model 1 vs Model 4	3.00(1)
Model 5: Model 1 + Paths between gc and job strain	467	912.35	0.06	0.96	0.96	Model 1 vs Model 5	0.01(1)
Model 6: Model 1 + Paths between gc and job engagement	467	912.35	0.06	0.96	0.96	Model 1 vs Model 6	0.01(1)
Model 7: Model 1 + Paths between gc and job satisfaction	467	908.25	0.05	0.96	0.96	Model 1 vs Model 7	4.11(1)*
Model 8: Model 7 + Paths between gc and intention to quit	466	876.31	0.05	0.96	0.97	Model 7 vs Model 8	36.05(1)***

N = 320. situ, situational issues; cult, organizational gender culture; inter, interpersonal issues; gc, glass ceiling; df, degrees of freedom; χ2, Minimum Fit Function Chi-Square; RMSEA, root-mean-square error of approximation; NNFI, Non-Normed Fit Index; CFI, Comparative Fit Index; and Δχ^2^, chi-square difference tests. *p < 0.05 and ***p < 0.001.

To evaluate whether this model offered the best depiction of our data, we successively added paths from each of the three antecedents of differential treatment to the glass ceiling (Models 2 to 4). However, these three latter models did not have a significantly better fit than Model 1. Starting with Model 1, we successively added paths from the glass ceiling to job strain (Model 5) and to job engagement (Model 6) but these latter two models did not have a significantly better fit than Model 1 [**Δ** χ^2^(1) = 0.01, *p* > 0.05]. We also added a path from the glass ceiling to job satisfaction (Model 7). This Model 7 presented a fit that was superior to Model 1 [**Δ** χ^2^(1) = 4.11, *p* < 0.05]. However, starting with this Model 7, we added a path from the glass ceiling to intention to quit (Model 8), and this latter model had a significantly better fit than Model 7 [**Δ** χ^2^(1) = 36.05, *p* < 0.001].

**FIGURE 2:**
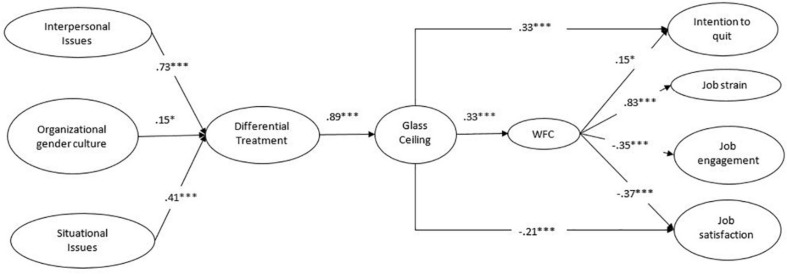
Completely standardized path coefficients for the retained model (Model 8); WFC, work-to-family conflict. For the sake of clarity, only structural relationships are shown. *p < 0.05 and ***p < 0.001.

Standardized parameter estimates for Model 8 are shown in Figure 2. For ease of presentation, we show the structural model rather than the full measurement model. Regarding our first hypothesis, situational issues, interpersonal issues and organizational gender culture were positively related to differential treatment. This latter concept was in turn positively associated with the glass ceiling. We used the bootstrapping technique to estimate indirect effects (Preacher and Hayes, 2008). As shown in Table 4, the indirect effects of situational issues, interpersonal issues and organizational gender culture on the glass ceiling through differential treatment were all significant. Thus, differential treatment totally mediates these relationships. These findings support Hypotheses 1a, 1b, and 1c.

**TABLE 4:** Indirect pathways using bootstrapping (hypotheses 1 and 2).

	Bootstrapping		Percentile 95% CI	
	Effect	SE	Lower	Upper
Indirect effect: x→m→y				
Situational issues→Differential treatment→Glass ceiling	0.1109	0.0378	0.0379	0.1863
Interpersonal issues→Differential treatment→Glass ceiling	0.2011	0.0323	0.1438	0.2744
0rganizational gender culture→Differential treatment→Glass ceiling	0.1711	0.0276	0.1227	0.2330

Glass ceiling→WFC→Job engagement	−0.0156	0.0095	−0.0410	−0.0014
Glass ceiling→WFC→Job strain	0.0450	0.0145	0.0203	0.0778
Glass ceiling→WFC→Job satisfaction	−0.0565	0.0190	−0.1009	−0.0254
Glass ceiling→WFC→Intention to quit	0.0361	0.0167	0.0109	0.0778

*N = 320. WFC, work-to-family conflict; SE, Standard Error; and CI, Confidence Interval; 10,000 bootstrap samples.*

Concerning our second hypothesis, results showed that the glass ceiling was positively associated with WFC, which in turn, was negatively related to job engagement and job satisfaction and positively related to job strain and intention to quit. The glass ceiling was also directly and positively related to intention to quit and negatively related to job satisfaction. As shown in Table 4, the indirect effects of the glass ceiling on outcomes through WFC were significant for all four outcomes (i.e., job strain, job engagement, job satisfaction, and intention to quit). Thus, WFC totally mediates the effects of the glass ceiling on job strain and job engagement, and partially mediates the effects of the glass ceiling on job satisfaction and intention to quit. These results totally support Hypotheses 2a and 2c, and partially support Hypotheses 2b and 2d.

Ancillary analyses were conducted in order to investigate whether differential treatment, the glass ceiling and WFC sequentially mediate the relationships. As indicated in Table 5, the indirect effects of the situational issues, interpersonal issues and organizational gender culture on the outcomes (i.e., job strain, job engagement, job satisfaction, and intention to quit) through differential treatment, the glass ceiling and WFC were all statistically different from zero. In sum, there is a triple mediation in sequence for all of these outcomes.

**TABLE 5:** Indirect pathways using bootstrapping (Ancillary analyses: triple mediation).

	Bootstrapping		Percentile 95% CI	
	Effect	SE	Lower	Upper
Indirect effect: x→m1→m2→m3→y				
Situational issues→Differential treatment→GC→WFC→Job engagement	−0.0016	0.0012	−0.0058	−0.0002
Situational issues→Differential treatment→GC→WFC→Job strain	0.0046	0.0025	0.0012	0.0119
Situational issues→Differential treatment→GC→WFC→Job satisfaction	−0.0056	0.0031	−0.0151	−0.0015
Situational issues→Differential treatment→GC→WFC→Intention to quit	0.0035	0.0023	0.0007	0.0112

Interpersonal issues→Differential treatment→GC→WFC→Job engagement	−0.0224	0.0110	−0.0475	−0.0034
Interpersonal issues→Differential treatment→GC→WFC→Job strain	0.0743	0.0328	0.0126	0.1410
Interpersonal issues→Differential treatment→GC→WFC→Job satisfaction	−0.0402	0.0155	−0.0751	−0.0135
Interpersonal issues→Differential treatment→GC→WFC→Intention to quit	0.0045	0.0033	0.0001	0.0137

Organizational gender culture→Differential treatment→GC→WFC→Job engagement	−0.0017	0.0015	−0.0065	−0.0002
Organizational gender culture→Differential treatment→GC→WFC→Job strain	0.0049	0.0032	0.0001	0.0123
Organizational gender culture→Differential treatment→GC→WFC→Job satisfaction	−0.0064	0.0040	−0.167	−0.0002
Organizational gender culture Differential treatment GC WFC Intention to quit	0.0039	0.0028	0.003	0.0125

*N = 320. GC, glass ceiling; WFC, work-to-family conflict; SE, Standard Error; CI, Confidence Interval; and 10,000 bootstrap samples.*

## Discussion

In this study, we try to better understand the phenomenon of the glass ceiling by considering both its antecedents and its possible consequences for well-being at work. More precisely, the present study has two objectives, allowing it to respond to a specific recommendation and to fill some gaps in the literature concerning this phenomenon of vertical discrimination against women in companies. Firstly, following the recommendation of Elacqua et al. (2009), we considered the organizational culture in relation to gender as a third factor (in addition to situational and interpersonal issues) in the emergence of the glass ceiling through the perception of differential treatment. (i.e., Hypothesis 1c). Secondly, we investigated the glass ceiling’s consequences for women managers’ organizational attitudes and well-being at work (i.e., Hypothesis 2). We did that to fill gaps, in particular with regard to relationships between the glass ceiling and work-family interface (i.e., WFC), and also because, to the best of our knowledge, no previous study has investigated the perception of WFC in association with the model proposed by Elacqua et al. These hypotheses were tested by asking women occupying managerial positions in a global healthcare company to self-report their perceptions.

Through our first Hypothesis, we postulated that perceptions of differential treatment will mediate the relationships between interpersonal issues, situational issues, organizational gender culture and the perception of a glass ceiling. Results indicated that interpersonal, situational issues and an organizational gender culture were all positively related to differential treatment, which was in turn positively related to the glass ceiling. As a reminder, we found no direct path from these three antecedents of differential treatment to the glass ceiling, suggesting that differential treatment fully mediates these relationships.

Results concerning interpersonal and situational issues are in line with previous findings (e.g., Elacqua et al., 2009; Cohen et al., 2020). On one hand, the lack of access to a mentor leads women to feel that they are excluded from communication and important opportunities for promotion (Allen et al., 2004). Moreover, as women are assigned positions with lower visibility, they have fewer opportunities to connect with high-ranking individuals and develop their social networks with senior managers (e.g., Ragins et al., 1998), reducing therefore their chances to exchange valuable strategic information, contacts and recommendations and, consequently, limiting their opportunities for promotion (Cohen et al., 2020). Lastly, by having fewer opportunities to develop friendly relationships with decision-makers, women managers have less chance of being promoted. These three interpersonal issues lead women mangers to perceive more differential treatment between them and their male colleagues, increasing therefore their perception of a glass ceiling within their organization.

On the other hand, two situational issues also influence the perception of differential treatment and ultimately the perception of a glass ceiling. Indeed, women considering that their company does not use objective criteria related to skills and performance for the promotion process rate the process as being unfair (Beehr et al., 2004), thus increasing their perception of a glass ceiling within their organization. Moreover, in observing that there are few women who have been in managerial positions long enough to be considered serious candidates for advancement to higher levels or that there are not as many women in a situation for promotion to higher-level management positions, women managers are more likely to conclude that they are treated differently than their male colleagues (Elacqua et al., 2009). Not feeling they are treated the same as their male counterparts, women managers will be more likely to perceive a glass ceiling within their organizations (Cohen et al., 2020).

However, in comparison to the study conducted by Elacqua et al. (2009), the added value of our research lies in the inclusion of a third factor in the emergence of differential treatment and ultimately the glass ceiling phenomenon. Indeed, the organizational culture in relation to gender within an organization can also increase women managers’ perceptions of being treated differently from their male counterparts. Working in an organization wherein women are excluded or/and marginalized, wherein gender equality policies are not applied or promoted, wherein gender stereotypes are common, women managers perceive a difference in the way they are treated and considered by the organization. When woman managers observe that their organization and/or their colleagues convey and encourage a “masculine” image of management and express, not necessarily consciously, negative stereotypes about their flexibility and mobility (e.g., van Vianen and Fisher, 2002; Broadbridge and Hearn, 2008; Hoobler et al., 2008; Kumra and Vinnicombe, 2010), they tend to consider that women and men are treated differently and that women managers are victims of a glass ceiling.

Concerning differential treatment, thinking that the performance of men and women is evaluated differently and that the distribution of rewards depends on gender (e.g., Ngo et al., 2002; Elacqua et al., 2009) increases women’s perceptions that some barriers inhibit their progression/advancement to higher positions in their organization.

Through our second Hypothesis, we postulated that WFC will mediate the relationships between the glass ceiling and job strain, intention to quit, job engagement, and job satisfaction. Results indicated that WFC was positively related to the glass ceiling, which was in turn positively related to job strain and intention to quit, and negatively related to job engagement and job satisfaction. We also found a direct positive relationship between the glass ceiling and intention to quit, and a negative one between the glass ceiling and job satisfaction. Therefore, these results suggest that WFC fully mediates the effects of the glass ceiling on job strain and job engagement, and partially mediates the effects of the glass ceiling on job satisfaction and intention to quit.

A glass ceiling reduces access to job resources (e.g., mentoring, important information/advice, social networks, opportunities to develop knowledge, and performance feedback) and undermines personal characteristics (e.g., self-esteem, self-efficacy, and optimism) and energies (e.g., knowledge, money) of women managers (e.g., Freeman, 1990; Powell and Butterfield, 1994; Baxter and Wright, 2000; Tran, 2014). In order to progress in the hierarchy despite obstacles related to the glass ceiling, women managers invest their limited resources (Hobfoll, 2011). However, in their attempts to override these almost insurmountable barriers, they deplete their resources. This situation ultimately results in a loss spiral in which more and more resources are lost (Hobfoll, 2001). The poor psychological and/or physical health emerging from this situation of a loss of resources negatively affects individuals’ functioning at home (Edwards and Rothbard, 2000), increasing women managers’ perceptions of WFC. Women managers perceiving WFC are more strained (e.g., Babic et al., 2015, 2019) due to the loss of resources in the process of juggling work and family roles (Hobfoll, 1989). In a such situation of WFC, they also are more likely to plan to leave their organization (e.g., Babic et al., 2015) and tend to reduce their level of engagement in their work (e.g., Babic et al., 2020) in order to preserve and protect their remaining resources (Hobfoll, 1989). Women managers psychologically attribute blame to the work domain and are dissatisfied with this domain given that it is the source of the conflict they perceive (Shockley and Singla, 2011). Therefore, when women managers perceive that their work interferes strongly and negatively with their family domain and causes difficulties in fulfilling family responsibilities, they are less satisfied with their job (e.g., Babic et al., 2015).

Our results also indicated that the glass ceiling was directly related to job satisfaction and intention to quit. Women managers who perceive a glass ceiling report being less satisfied with their job and having more intention to quit. These effects could be explained by the lack of career advancement opportunities they have/perceive when facing such a situation of vertical discrimination (e.g., Nelson et al., 1990; Stroh et al., 1996; Foley et al., 2002).

### Limitations, Strengths, and Future Perspectives

Our study has some limitations leading to interpreted findings with caution. The major limit of our study concerns the cross-sectional design of our research. Indeed, such a design precludes any inference of causality among the variables. Therefore, the present study should be replicated by using longitudinal designs with repeated measures to investigate the direction of causality between the variables included in our model. Another limitation refers to the fact that data are based on self-reported measures. Indeed, our study focuses on women managers’ perceptions. However, using self-reported data may have reduced the validity of our results. Indeed, this kind of data can produce two important biases. The first one concerns the social desirability influence bias and the second refers to the common method bias (Podsakoff et al., 2012). Nevertheless, in order to counter such bias, we followed several recommendations and took several precautions at both methodological and statistical levels. On one hand, at the methodological level, participants were assured that their responses were anonymous and confidential and that there were no right or wrong answers to the questions. We also used largely validated questionnaires to assess the glass ceiling’s consequences for organizational attitudes and well-being, and performed confirmatory factor analyses to demonstrate their validity. On the other hand, at the statistical level, we performed the Harman’s single-factor test (Podsakoff et al., 2012). Results indicated that the common method bias was not a major threat to our results. Although we included age, grade and presence of children at home as covariates, our model could be influenced by other socio-demographic variables. Indeed, the age of the youngest child, for example, influences the perception of WFC (e.g., Byron, 2005). It is therefore impossible for us to guarantee that the relationships we studied have been isolated from spurious influences (Bollen, 1989). As our data were collected in one specific organization, it is also difficult to generalize our results. Moreover, the type of sector (i.e., public or private) seems to influence the perception of the glass ceiling. Indeed, Sever (2016) found that those who worked in private sectors claimed that they felt a glass ceiling effect more than those who worked in the public sector. As we conducted our study within a private company, it would be interesting to replicate it with other samples coming from the public sector.

Although we are aware of these limitations, our study also has several strengths. The first is that we tend to better understand the phenomenon of the glass ceiling by considering both its antecedents and its possible consequences for well-being at work. We extend the model developed by Elacqua et al. (2009) by proposing a more comprehensive model including the culture in relation to gender within the organization. We also investigated the impact of the glass ceiling on women managers’ organizational attitudes and well-being at work by considering WFC as a possible mediator. In doing so, our study contributes to the literature considering that, to the best of our knowledge, no previous research has investigated the relationship between the glass ceiling and WFC through the model developed by Elacqua et al. (2009). Our findings are consistent with theories/models that are largely recognized in the literature (e.g., the COR model, Hobfoll, 1989, 2001, 2011; and the spillover theory, Edwards and Rothbard, 2000).

Having said that, our study may have gone one step further. Through the present study, we focused only on the negative side of the work-family interface (i.e., WFC). However, over the last years, there has been a move to understand and examine the potential benefits of occupying multiple roles. Researchers refer to this positive side of the work-family interface by often employing the term of work-family enrichment defined as the “extent to which experiences in one role improve the quality of life in the other role” (Greenhaus and Powell, 2006, p. 73). Therefore, it would be interesting to include the positive side of work-family interface in the present study in order to have a comprehensive understanding of the links between glass ceiling and work-family interface. Indeed, considering that conflict and enrichment coexist (e.g., Grzywacz and Butler, 2005; Rantanen et al., 2013), focusing only on one of the two sides limits our understanding of the underlying processes (Boz et al., 2016).

Furthermore, as developed earlier, resources seem to play an important role in the relationship between the process of the glass ceiling, WFC and well-being, as evidenced by, for example, the conservation of resources theory detailed throughout this research. Therefore, to understand the underlying mechanism more fully, future research should investigate the role of resources or affects as mediators in these relationships.

### Practical Implications

Our results suggest that the glass ceiling may have deleterious effect on work-family conciliation, on well-being at work (i.e., job strain and job engagement) and on organizational attitudes (i.e., intention to quit and job satisfaction). Our study also highlights the importance of all three factors (i.e., the interpersonal, situational issues, and organizational gender culture) in the emergence of the perception of differential treatment by gender within an organization and ultimately to the perception that a glass ceiling exists. Therefore, one way to break down women managers’ perceptions of being differently treated from their male colleagues is to act on these three factors. Indeed, organizations/employers could adopt some strategies or practices to eliminate (or at least reduce) the perception of the glass ceiling. Of course, women themselves are also essential players in breaking down barriers related to this vertical discrimination and its consequences for WFC, well-being and organizational attitudes.

From the interpersonal issues perspective, it will be important to give women managers access to a mentor in order to include them in important communication and to exchange valuable strategic information, contacts and recommendations. Indeed, mentoring programs are one of the most effective ways to avoid barriers related to the glass ceiling and move into top management positions (Dworkin et al., 2012). Thus, organizations have to provide a strong mentoring program. Failing that, women have to be proactive in finding a senior level (female) mentor (within the organization or not) who has faced and successfully overcome similar obstacles and challenges (e.g., Ragins and Cotton, 1999). Moreover, organizations can also give women managers a more visible position by allowing them to develop friendly relationships with decision-makers and connect with high-ranking individuals and improve their social networks with senior managers. In other words, organizations have to provide sufficient support for women’s career progression and to implement/promote strategies creating interpersonal communication and networks (Ohemeng and Adusah-Karikari, 2015).

From the situational issues perspective, organizations have to use objective criteria related to skills and performance in the promotion process. It is also important that employers place women managers in important developmental positions, hire more women for high-level management positions and give them opportunities to develop their skills and competences to move more readily to the top of the hierarchy. Organizations have to invest in training and support specifically designed for women, including career and leadership development programs (Kassotakis, 2017; Broadbridge and Fielden, 2018). In benefiting from such programs, women have the possibilities to develop their leadership competences in particular. Human resource policies have to encourage women to advance or be promoted just as men are. In so doing, women are more likely to be considered serious candidates for advancement to higher levels within their organization.

Lastly, from the organizational gender culture perspective, organizations have to apply/promote gender equality policies, especially in order to avoid women feeling excluded or/and marginalized. It is also crucial to combat gender stereotypes by, for example, maintaining some strict rules and regulations against adverse unprofessional acts (Bell et al., 2002). Organizations could also implement sensitization programs about the glass ceiling in order that all employees learn and adopt the values of respect for all, social knowledge and expected good behaviours (Haynes and Ghosh, 2011).

Given all of these (non-exhaustive) strategies or practices, breaking through the glass ceiling is complex and requires action on several fronts but it is not an insurmountable task. However, as mentioned by du Plessis et al. (2015, p. 45), “practices that facilitate gender equality and the removal of the glass ceiling might increase the cost for organizations, but in the long run the pros will definitely outweigh the cons. The better the employees are treated, the higher profits and productivity that organizations have in return as job satisfaction is one of the most crucial factors to improve employees’ performance and organizations’ performance as a whole.”

## Data Availability Statement

The raw data supporting the conclusions of this article will be made available by the authors, without undue reservation.

## Ethics Statement

Ethical review and approval was not required for the study on human participants in accordance with the local legislation and institutional requirements. The patients/participants provided their written informed consent to participate in this study.

## Author Contributions

AB was the main project leader and IH was the supervisor of the project. Both authors contributed to the article and approved the submitted version.

## Conflict of Interest

The authors declare that the research was conducted in the absence of any commercial or financial relationships that could be construed as a potential conflict of interest.

## Footnotes

1 https://statbel.fgov.be/fr/visuals/travail-et-genre

2 https://www.lecho.be/entreprises/chimie/trois-femmes-ceo-dans-le-bel-20/10057204.html
