# Supplementary material for: The Glass Ceiling for Women Managers: Antecedents and Consequences for Work-Family Interface and Well-Being at Work
Source: Front Psychol. 2021 Mar 9;12:618250. doi: 10.3389/fpsyg.2021.618250 (PMC7985459; doi:10.3389/fpsyg.2021.618250)
Supplement: Supplementary file 1 [file Data_Sheet_1.pdf]

# APPENDIX 1

Items used to assess the glass ceiling and its antecedents. Items developed by Elacqua, Beehr, Hansen and Webster (2009) are in bold.

<u>Interpersonal issues</u>

**1. I regard my immediate manager as my mentor.**
**2. I would guess most male managers at my company feel they are part of an informal senior managers' network.**
**3. Most male managers tend to think alike and have similar personal interests.**
**4. Having a personal friendship with the decision-makers determines whether an employee will be considered for a promotion at my company.**
5. Women managers have less opportunities of accompaniment by a more experimented manager.
6. Few women managers belong to an informal senior managers' network.
7. Few women managers have access to strategic information coming from the senior management.
8. Women manager tasks do not allow them to show and make themselves known to the senior management.

<u>Situational issues</u>

**1. Establish objective and standard hiring/promotion criteria would increase the likelihood that women will advance in my company.**
**2. There are few women at the top rung of management at my company because they haven't been in the management ranks long enough.**
**3. There aren't many women in senior management positions at my company because women with executive potential have not been in the 'pipeline' long enough.**
4. In my company, the selection procedure is based on objective criteria.
5. In my company, the accession to formations is done in terms of objective criteria.
6. In my company, promotion procedure is based on objective criteria.

<u>Differential treatment</u>

**1. My company spends as much time and money to develop women as it does to develop men.**
**2. Female managers at my company make less than their male manager counterparts.**
**3. At my company, there are differences in salaries and titles for men and women in the same positions that are not explained by differences in performance, education, experience.**
**4. Create special career development programs for women would increase the likelihood that women will advance in my company.**
**5. Establish a 'fast track' promotion program for women would increase the likelihood that women will advance in my company.**
6. In my company, performance criteria are different for men and women managers.
7. In my company, tasks assigned to men and women managers are different.

<u>Organizational gender culture</u>

1. My colleagues and superiors estimate that women managers are less mobile and flexible than their male counterparts.

2. My colleagues and superiors estimate that the conflict between professional and family life is more present among women managers than their male counterparts.
3. My company conveys the image of an authoritarian, aggressive, dominant manager.
4. In my company, behaviors defined as “masculine” (authority, aggressiveness, …) are encouraged amongst the managers.

<u>**Perceptions of a glass ceiling**</u>

**1. Female managers/ supervisors at my company generally progress to a certain level, then go no further.**
**2. I believe my company is serious about eliminating barriers that prevent women from reaching their potential.**
**3. Female managers/supervisors at my company are often excluded from important senior management communications.**
4. In my company, male managers are more rapidly promoted than women managers.
5. In general, women managers are treated equally in my company.
6. In my company, with equal experience and expertise, men have access to higher positions in the hierarchy than women.
