# Supplementary material for: The Glass Ceiling for Women Managers: Antecedents and Consequences for Work-Family Interface and Well-Being at Work
Source: Front Psychol. 2021 Mar 9;12:618250. doi: 10.3389/fpsyg.2021.618250 (PMC7985459; doi:10.3389/fpsyg.2021.618250)
Supplement: Supplementary file 2 [file Image_1.pdf]

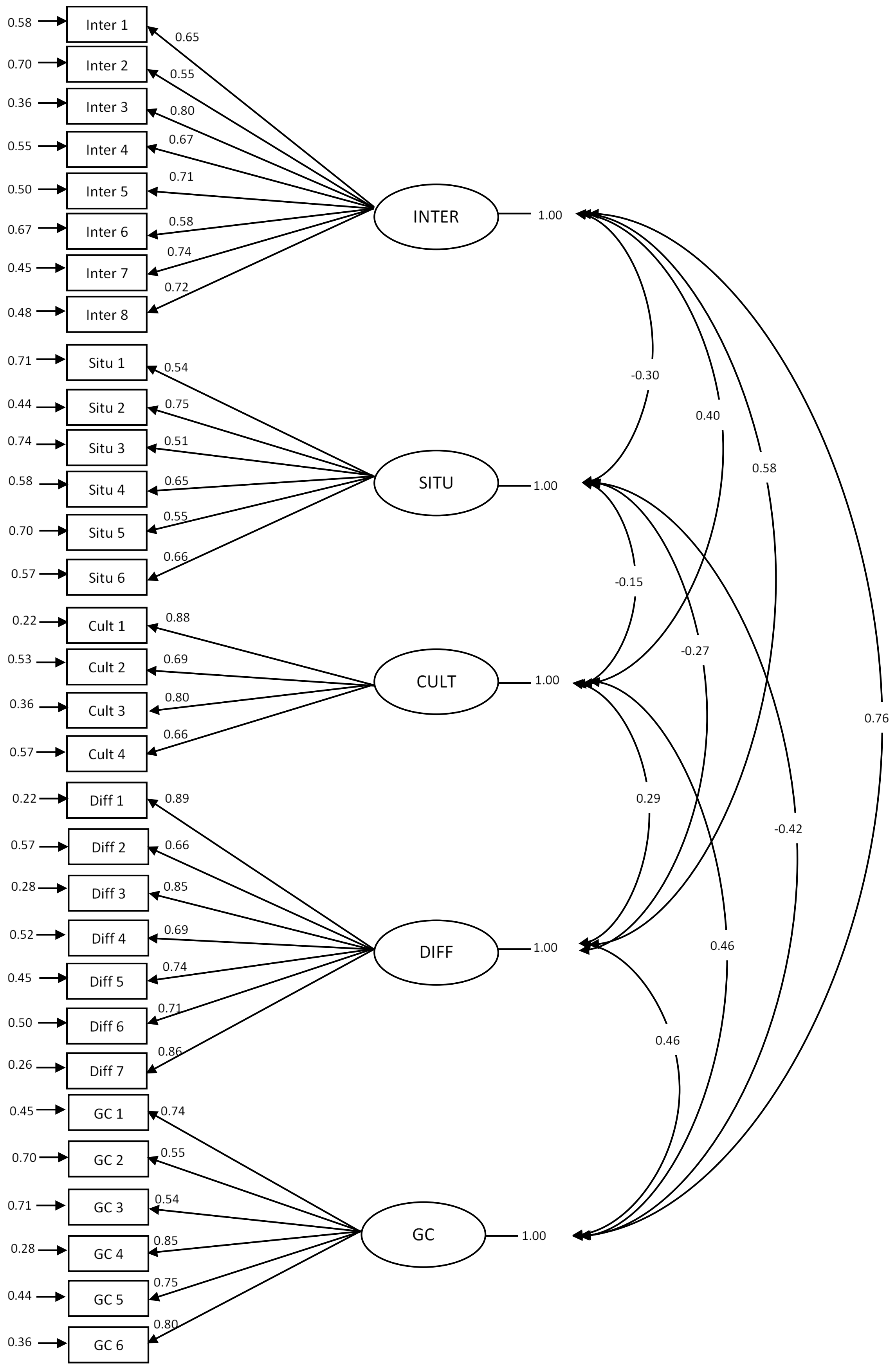

Figure 1. Full measurement model.
INTER= Interpersonal issues; SITU= Situational issues; CULT= Organizational gender culture; DIFF= Differential treatment; GC= Perceptions of a glass ceiling
